# Supplementary material for: Effect of Strain Rate on Single Tau, Dimerized Tau and Tau-Microtubule Interface: A Molecular Dynamics Simulation Study
Source: Biomolecules. 2021 Sep 4;11(9):1308. doi: 10.3390/biom11091308 (PMC8472149; doi:10.3390/biom11091308)
Supplement: Supplementary file 1 [file biomolecules-11-01308-s001.zip › biomolecules-1366665-supplementary.pdf]

biomolecules

MDPI

Article

Effect of Strain Rate on Single Tau, Dimerized Tau and Tau-Microtubule Interface: A Molecular Dynamics Simulation Study

Md Ishak Khan [1], Kathleen Gilpin [2], Fuad Hasan [1], Khandakar Abu Hasan Al Mahmud [1] and Ashfaq Adnan [1,*]

[1] Department of Mechanical and Aerospace Engineering, University of Texas at Arlington, Arlington, TX 76019, USA; mdishak.khan@mavs.uta.edu (M.I.K.); fuad.hasan@mavs.uta.edu (F.H.); mahmud.khandakarabuhas@mavs.uta.edu (K.A.H.A.M.)

[2] Academic Partnership and Engagement Experiment (APEX), Wright State Applied Research Corporation, Beavercreek, OH 45431, USA; kathleen.gilpin@navy.mil

* Correspondence: aadnan@uta.edu

Supplementary Material

Supplementary Material Part 1

Reliability, Applicability, and Limitation of the Predicted Tau Structure

1.1 i-TASSER Methodology:

In the manuscript, we have studied the single tau, dimerized tau, and tau-MT interaction. The structure of tau protein which is an intrinsically disordered protein (IDP) is obtained by using predictor software i-TASSER [1]. i-TASSER uses a hierarchical protein structure modeling approach based on the secondary-structure enhanced Profile-Profile threading Alignment (PPA) [2] and the iterative implementation of the Threading ASSEmbly Refinement (TASSER) program [3]. In order to address the problem of predicting 3D structures of proteins in structural biology and completely automate the system by computer algorithm while admitting the importance of intervention of human expert, i-TASSER has set a methodology to obtain reliable predicted structure for protein with appropriate quantification of the quality of the models. Due to the reliability of its predicted structure, i-TASSER has been ranked as the best method in the server section of the 7th CASP (Critical Assessment of Structure Prediction) experiment [4].

The scoring function (C-score) of this software, is based on the relative clustering structural density and the consensus significance score of multiple threading templates. In order to validate the reliability of the scoring system, the developer group of the software has performed a large benchmark test on 800 non-homologous single domain random proteins, which demonstrates a strong correlation between the C-score and the TM-score (a structural similarity measurement with values in [0, 1]) of the first models with a

correlation coefficient of 0.91. Using a C-score cutoff > -1.5 for the models of correct topology, both false positive and false negative rates are below 0.1. Combining C-score and protein length, the accuracy of the I-TASSER models can be predicted with an average error of 0.08 for TM-score and 2 Å for RMSD.

Although we have cited the paper which describes the quantitative measure i-TASSER takes to ensure the model reliability, we are briefly discussing the C-score and TM-score here for relevance.

The C-score is defined as:

$$\mathbf{C-score = \ln\left(\frac{M}{M_{total}} \times \frac{1}{\langle RMSD\rangle} \times \frac{\prod_{i=1}^{4} Z(i)}{\prod_{i=1}^{4} Z_0(i)}\right)}$$

where M is the multiplicity of structures in the SPICKER cluster [5]; M_{tot} is the total number of the i-TASSER structure decoys used in the clustering; ⟨RMSD⟩ is the average RMSD of the decoys to the cluster centroid; Z(i) is the highest Z_{score} (the energy to mean in the unit of standard deviation) of the templates by the ith PPA threading program and $Z_0(i)$ is a program-specified Z-score cutoff for distinguishing between good and bad templates. The first two factors of the equation accounts for the degree of structure convergence of the SPICKER cluster, while the third factor accounts for the quality of the threading alignments. The logarithm is taken to ensure the even adjustment of the distribution of the C-score. The previous definition of C-score proved the strong correlation between the C-score and the reliability of the predicted models [4], while the definition of C-score is slightly different due to the normalization of Z-score, which extends the scope of the definition to the cases where different threading algorithms might be used to predict the same template. Furthermore, it accounts for the consensus of the alignment confidence of multiple threading programs.

The second scoring function, the TM-score [6] is defined as:

$$\mathbf{TM-score = \frac{1}{L}\sum_{i=1}^{L} \frac{1}{1+\frac{{d_i}^2}{{d_0}^2}}}$$

where d_i is the distance of the ith pair of residues between two structures after an optimal superposition, $\mathbf{d_0 = 1.24\sqrt[3]{L-15} - 1.8}$, and L is the protein length. TM-score stays in [0, 1] with higher values indicating better models. Statistically, a TM-score ≤ 0.17 corresponds to a similarity between two randomly selected structures from the PDB library; a TM-score > 0.5 corresponds

approximately to two structures of the similar topology. One advantage of the TM-score is that the meaning of the TM-score cutoffs is independent of the size of proteins.

1.2 Predicted Structure of Tau Protein from i-TASSER:

After submitting the tau protein amino acid sequence to i-TASSER queue, we have obtained 5 predicted “pdb format” models as output. For each target, i-TASSER simulations generate a large ensemble of structural conformations, called decoys. To select the final models, i-TASSER uses the SPICKER program to cluster all the decoys based on the pair-wise structure similarity and reports up to five models which corresponds to the five largest structure clusters. The confidence of each model is quantitatively measured by C-score that is calculated based on the significance of threading template alignments and the convergence parameters of the structure assembly simulations. C-score is typically in the range of [-5, 2], where a C-score of a higher value signifies a model with a higher confidence and vice-versa. TM-score and RMSD are estimated based on C-score and protein length following the correlation observed between these qualities.

According to i-TASSER policy, they report C-score for all the five models but provide TM-score and RMSD values for only the first one (the most reliable model in most cases, because of the usage of the biggest cluster for the first model). Fig. S1 shows the five models they have reported.

The first model is used for our simulation, which has the C-score of -0.03, estimated TM-score = 0.71±0.12, and estimated RMSD = 7.1±4.2Å. As it is at the very reliable end of the C-score, and the TM score is far higher than >0.5 which ensures a good topology, we have proceeded with the reported structure. We already know that tau protein is an intrinsically disordered protein (IDP), and there can be multiple stable conformations. However, by strictly following the reliability parameters defined by i-TASSER, we can proceed with the first predicted model.

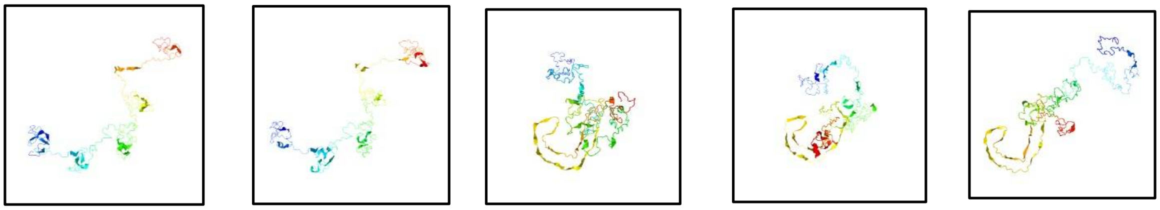

a

b

c d e

Figure S1: Predicted tau protein structure models from i-TASSER. a. Model 1: C-score = -0.03, estimated TM-score = 0.71±0.12, and estimated RMSD = 7.1±4.2Å (used for our simulations), b. Model 2: C-score = -0.03, c. Model 3: C-score = -3.57, d. Model 4: C-score = -3.14, e. Model 5: C-score = -3.91. The color coding is blue to read, meaning from C-terminal to N-terminal.

1.3 Limitations of the Prediction of Tau, which is an IDP:

The authors of this manuscript are completely aware that it is intuitive to question the reliability of the predicted structure of an IDP. However, to pave the pathway to characterize the material properties of tau protein, we must compromise between the available experimental repertoire and the convenience that the computational approaches provide. Experimentally, the research groups have tended to determine the 3D structure of tau protein of only diseased tau proteins by dint of cryo-EM procedures, which facilitated the development of structure and availability of diseased tau protein in the Protein Data Bank (PDB). It has been possible to obtain the filamentous structure of tau protein for chronic traumatic encephalopathy (CTE) [7], pick's disease [8], or Alzheimer's disease (AD) [9] affected tau protein, which are in general formed by the result of abundance of hyperphosphorylation sites, or repeated impact on the head leading to neuropathology. From the experimental data in the literature, the filamentous structure, paired helical filaments (PHF), or neurofibrillary tangles (NFT) are only formed in diseased tau protein and/or their aggregates. To find the mechanical properties, we must proceed with normal (healthy) tau protein structure, which is not yet experimentally determined. Even the partial structures of diseased tau proteins are made available in the PDB very recently [7,10] (in the years of 2019-2020, prior to which our study begun, and we have continued to review the literature to cope up with the very recent advancement of protein structure determination).

Therefore, our computational approach has addressed certain aspects of tau protein that have not yet been addressed by any experimental means to date, according to the best knowledge of the authors. Furthermore, we have justified the reliability of the predicted structure in this supplementary material. The limitation of this work is that due to computational constraint, other conformations of tau protein which are competitively reliable from the perspective of C-score and TM-score have not been statistically explored. However, we plan to address this issue as well in the continuing study. We admit that there are certain unanswered areas in the study of tau protein which are not revealed by experimental means conclusively to date (such as the mechanism of phosphorylation in altering the properties of tau protein, which we plan to address in our continuing study), and based on the recent molecular level tau studies, we can proceed with our representative structure of tau, which can be suggested as "one possible conformation which is stable with reliable mark of confidence". In the main manuscript, we have cited some relevant tau studies which have used i-TASSER predicted structure.

Supplementary Material Part 2

Simulation in Explicit Solvent vs Implicit Solvent for Tau Protein and Tau-MT Interaction

A molecular dynamics simulation can be carried out in two ways – with explicit solvation or implicit solvation. In explicit solvation, the simulation box is filled with explicit water molecules, whereas in case of implicit solvation, the interaction between the molecule

of concern and implicit water molecules is defined analytically. The definitions of such interactions have been defined by establishment of a handful of models e.g. models which consider mean potential force [11], or models which consider distance-dependent electrostatic interaction [12]. Aside from the established importance of explicit water molecule solvation which is closer to the realistic physical phenomenon, it is relevant to discuss the validity and reliability of using implicit solvation technique. A direct study comparing the effect of the solvent molecule type on the mechanical behavior of tau protein is not currently available in the literature. However, this supplementary material is an attempt to explain the expected difference between the two types of simulations.

In this study, explicit solvation has been used for single tau simulations, but implicit solvation was used for dimerized tau and tau-MT simulations due to the bigger box size involved. Using implicit solvent techniques in some of the simulations consisted adjustment of coulombic terms by modifying 1/r terms to $1/r^2$. The advantages for this method are two-fold, such as:

1. Ability to ignore Ewald sum or potential mean force type interaction,
2. Accommodating large deformation associated with the applied high strain rate.

While these provide certain level of conveniences while simulating mechanical response of the respective axonal cytoskeletal components of neuron at high stretch, the solvent-molecule surface charged layer interactions are being ignored. Keeping these in consideration, differences can be expected in the calculated stress values, because:

1. The initial length of the proteins in the two systems will not be equal,
2. In explicit system, the tau protein will be stretched against resistance of solvent molecule interaction,
3. Incremental stretch will expose more charged [13–15] and hydrophobic surface [16] of the flanking (projection) domain of tau protein in case of explicit system, unlike in implicit system,
4. Solvent accessible surface area will continuously change as the protein unfolds and moves in the box during equilibration and tensile test. Note that the projection domain and the tail domain are not constrained in space,
5. Irrespective of the box orientation and filament orientation, the length scale of tau protein will be always significantly smaller than the box size due to the inherent disordered portion [16] of tau protein structure, and therefore, explicit water molecule interaction cannot be ignored.

Considering the abovementioned differences, it is expected that the stress development will be significantly higher in case of tau protein stretching in explicit solvent than that in implicit solvent, meaning implicit solvation will undercalculate the development of stress.

Now, as a direct comparison between the explicit and implicit equilibration and tensile tests that are performed to obtain an insight regarding the difference in stress-strain relation and the energy terms, a comparison test is also performed for single tau. The comparison test equilibrates two systems with the same initial structure of tau protein, equilibrates in the same condition, and then performs tensile test at $1x10^8 s^{-1}$ strain rate up to 80% strain of the projection domain. The objective is to compare the structural and energetical differences that occur during the comparison test.

The equilibration and tensile test are completed in two steps:

1. Equilibration for 100ps at 1fs timestep to equilibrate the system adequately,

2. Performing the tensile test by fixing the MT binding region and pulling the projection domain towards the -x direction up to 80% strain.

It is to be noted that in the implicit solvation, the system contains only 6424 atoms of the protein, while for the explicit solvation, it contains 410197 atoms in total. The appropriate density of water is maintained automatically by CHARMM-GUI.

Below, the steps will be detailed with appropriate data analysis and snapshots.

Equilibration and Tensile Test Comparison

Step 1: Equilibration for 100ps with 1fs timestep

Figure S2 shows the comparison between potential energy graphs in the explicit and implicit system. Figure S3 shows the Snapshots of the initial, intermediate, and final snapshot of the protein structure.

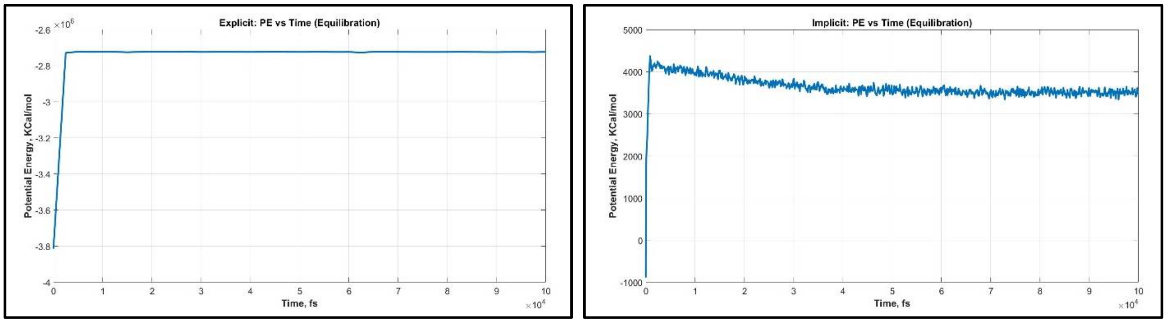

a

b

Figure S2: Equilibration: Potential energy vs time for a) explicit solvation, b) implicit solvation. Both systems are well-equilibrated, as both the graphs show that potential energy changes negligibly over time.

0ps 50ps 100ps

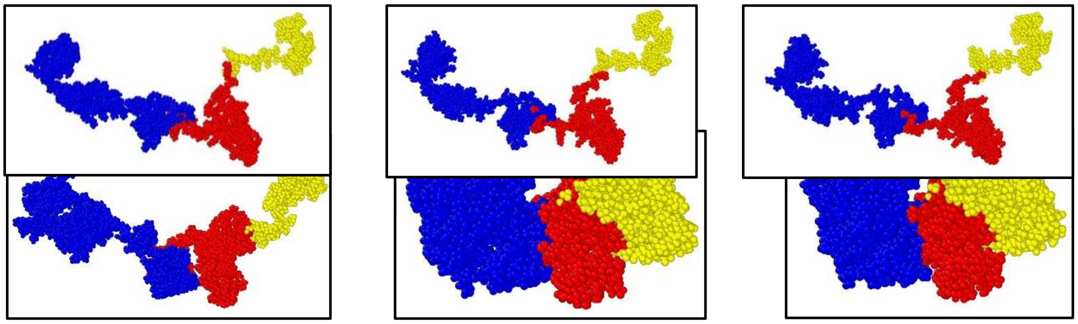

a

b

c

d e f

Figure S3: Equilibration: Structural snapshot of the tau protein for a-c) explicit solvation, d-f) implicit solvation at left: 0ps, middle: 50ps, right: 100ps. Legend: Blue: projection domain, Red: MT binding sites including interrepeats, Yellow: tail.

As Figure S3 depicts, the major structural difference occurs over this 100ps equilibration. Due to the continuous interaction with explicit water molecules, the projection domain retains its expanded structure (expands ~2Å by the end of the 100ps equilibration, meaning no unfolding or significant structural change occurs) for explicit solvation, while due to continuous interaction between the positively and negatively charged region, the projection domain shrinks on itself, or "tightens" the existent folds. At the end of the 100ps equilibration, the length of the projection domain (determined by the difference of the highest and lowest x coordinate of atom in the projection domain) becomes significantly smaller, ~39Å, which is ~57.6% shrinkage. This leads to a significant difference of the initial length for the tensile test.

Step 2: Performing the tensile test by fixing the MT binding region and pulling the projection domain towards the -x direction up to 80% strain

Figure S4 shows the comparison between stress vs strain in the explicit and implicit system. Finally, Figure A2.5 shows the Snapshots of the protein structure at every 8% incremental strain for insight of unfolding phenomenon.

Explicit Implicit

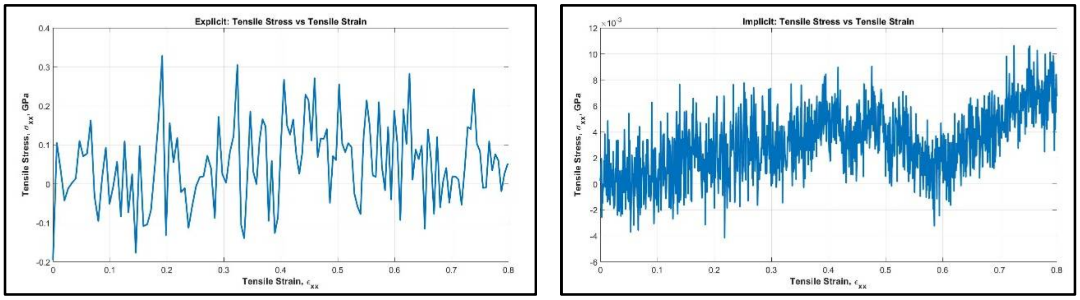

b

a

Figure S4: Tensile test: Stress vs strain for a: explicit system and b: implicit system.

From the PDB file cartoon representation (Figure S5) it is evident that there are three major tertiary folded regions in the projection domain: 1) Residue 1-80 containing one α-helix in between, 2) Residue 87-160 containing another α-helix in between, and c) Residue 187-242. For convenience, these regions will be called as regions A, B, and C afterwards in this section.

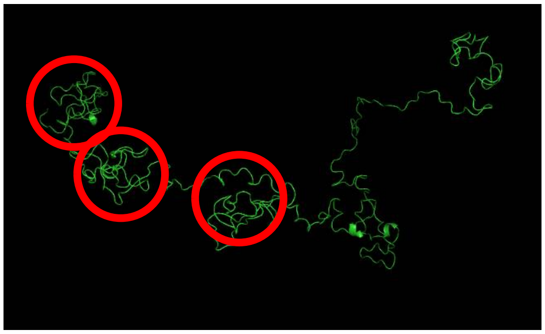

Figure S5: Cartoon representation of the PDB file of the initial structure. Regions A, B, and C are marked by red circles. Visualization by PyMol [17].

While the tension is applied towards the -x direction, simultaneous unfolding begins to take place in these three regions. It is important to verify that both the systems with explicit and implicit solvation can capture similar trend of unfolding. Furthermore, the intermediary portions in between the regions A, B, C will be also unfolded or stretched, and these are to be considered as well. However, the folding situation is significantly different in case of implicit system, as the whole protein is shrunk on itself. To make a direct comparison, the gradual evolution of the equivalent portions will be tabulated.

Explicit Implicit

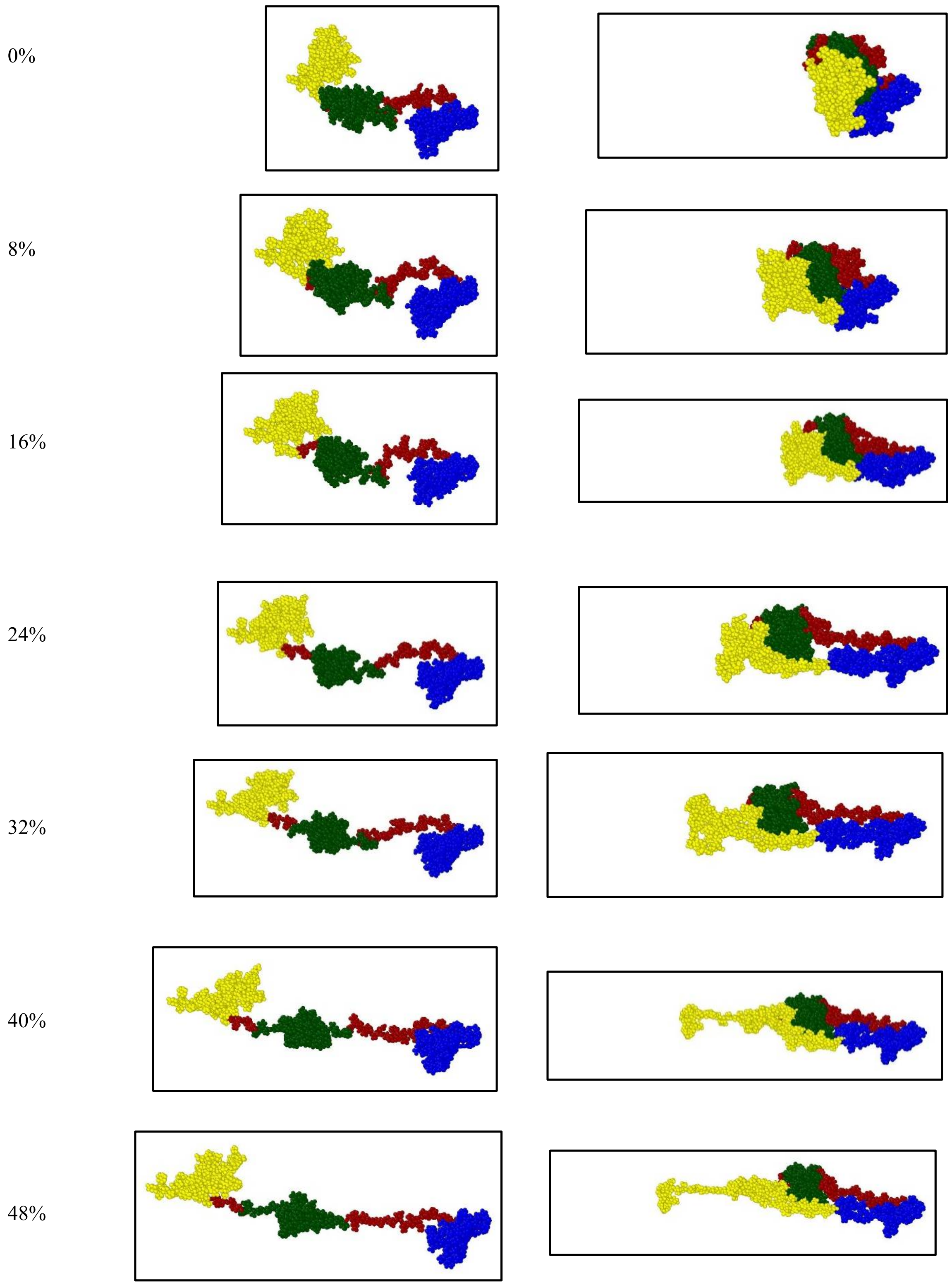
0%
8%
16%
24%
32%
40%
48%

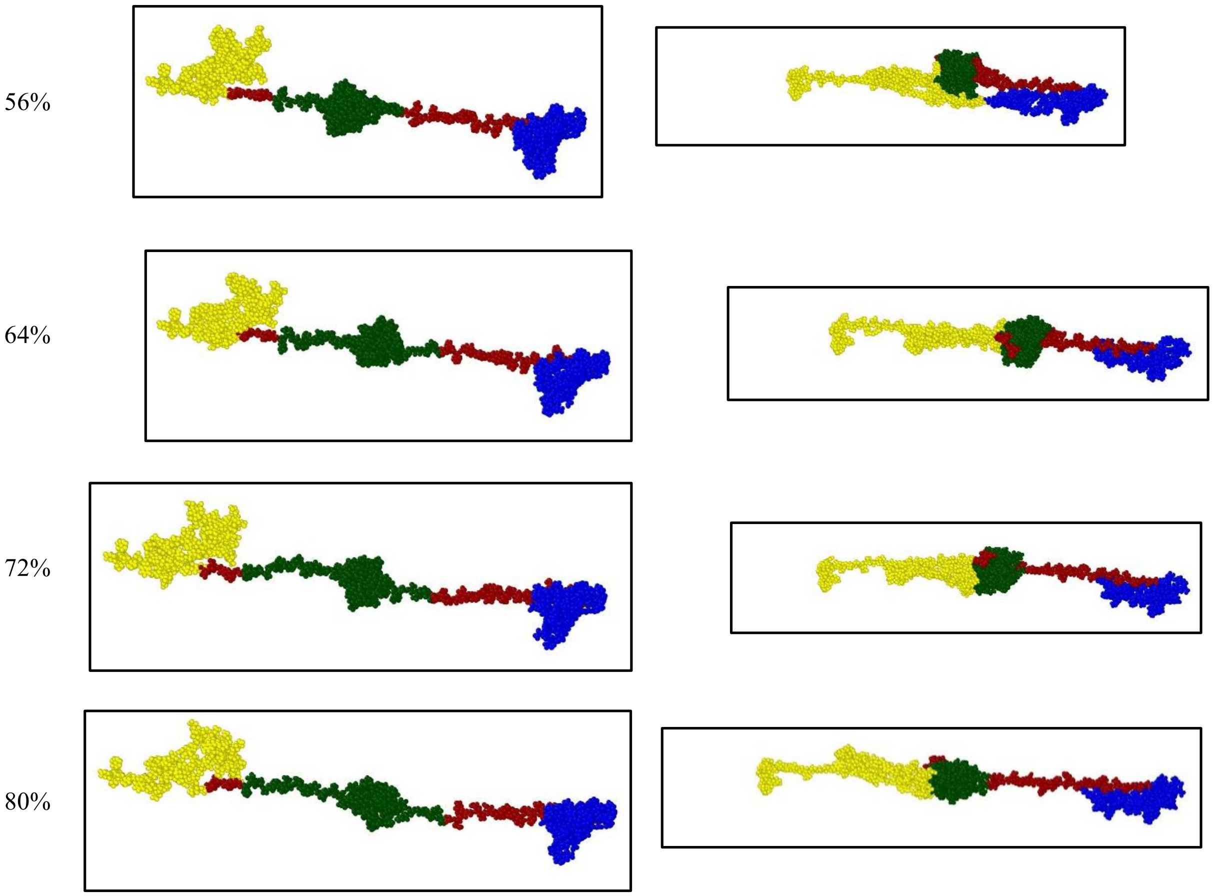

Figure S6: Tensile test: Structural snapshot of the tau protein for Left: explicit solvation, Right: implicit solvation. For the convenience of visualization, only the projection domain is shown. Legend: Yellow: First major fold, Green: second major fold, Blue: third major fold, Maroon: intermediary region between the major folded regions.

Now, Table S1 below summarizes the unfolding phenomena that take place for explicit and implicit solvation.

Table S1: Gradual Progression of Unfolding of Single Tau

Strain	Observation: Explicit	Observation: Implicit
0%	Initial	Initial
8%	A: initialization of unfolding,<br><br>B: unfolding in multiple places,	A: initialization of unfolding,<br><br>B: initialization of unfolding,

	C: initialization of unfolding,<br><br>Between A and B: straightening,<br><br>Between B and C: straightening.	C: initialization of unfolding,<br><br>Between A and B: initialization of unfolding,<br><br>Between B and C: initialization of unfolding.
16%	A: visible unfolding,<br><br>B: continued unfolding in multiple places,<br><br>C: initialization of unfolding,<br><br>Between A and B: visible unfolding,<br><br>Between B and C: visible unfolding.	A: initialization of unfolding,<br><br>B: initialization of unfolding,<br><br>C: initialization of unfolding,<br><br>Between A and B: initialization of unfolding,<br><br>Between B and C: visible unfolding.
24%	A: visible unfolding,<br><br>B: visible unfolding,<br><br>C: initialization of unfolding,<br><br>Between A and B: visible unfolding,<br><br>Between B and C: visible unfolding.	A: visible unfolding,<br><br>B: initialization of unfolding,<br><br>C: initialization of unfolding,<br><br>Between A and B: initialization of unfolding,<br><br>Between B and C: significant unfolding.
32%	A: visible unfolding,<br><br>B: visible unfolding,<br><br>C: initialization of unfolding,<br><br>Between A and B: significant unfolding,<br><br>Between B and C: significant unfolding.	A: significant unfolding,<br><br>B: initialization of unfolding,<br><br>C: significant unfolding,<br><br>Between A and B: initialization of unfolding,<br><br>Between B and C: significant unfolding.
40%	A: significant unfolding,<br><br>B: significant unfolding,<br><br>C: visible unfolding,<br><br>Between A and B: significant unfolding,<br><br>Between B and C: significant unfolding.	A: significant unfolding,<br><br>B: visible unfolding,<br><br>C: visible unfolding,<br><br>Between A and B: visible unfolding,<br><br>Between B and C: significant unfolding.

48%	A: significant unfolding,<br>B: significant unfolding,<br>C: significant unfolding,<br>Between A and B: significant unfolding,<br>Between B and C: significant unfolding.	A: significant unfolding,<br>B: visible unfolding,<br>C: visible unfolding,<br>Between A and B: visible unfolding,<br>Between B and C: significant unfolding.
56%	A: significant unfolding,<br>B: significant unfolding,<br>C: significant unfolding,<br>Between A and B: significant unfolding,<br>Between B and C: significant unfolding.	A: significant unfolding,<br>B: significant unfolding,<br>C: significant unfolding,<br>Between A and B: visible unfolding,<br>Between B and C: significant unfolding.
64%	A: significant unfolding,<br>B: significant unfolding,<br>C: significant unfolding,<br>Between A and B: significant unfolding,<br>Between B and C: significant unfolding.	A: significant unfolding,<br>B: significant unfolding,<br>C: significant unfolding,<br>Between A and B: significant unfolding,<br>Between B and C: significant unfolding.
72%	A: significant unfolding,<br>B: significant unfolding,<br>C: significant unfolding,<br>Between A and B: significant unfolding,<br>Between B and C: significant unfolding.	A: significant unfolding,<br>B: significant unfolding,<br>C: significant unfolding,<br>Between A and B: significant unfolding,<br>Between B and C: significant unfolding.
80%	A: significant unfolding,<br>B: significant unfolding,<br>C: significant unfolding,	A: significant unfolding,<br>B: significant unfolding,<br>C: significant unfolding,

	Between A and B: significant unfolding,<br><br>Between B and C: significant unfolding.	Between A and B: significant unfolding,<br><br>Between B and C: significant unfolding.

From Table S1 it is evident that the unfolding mechanism differs between the explicit and implicit system for up to 56% strain which can be attributed to the difference in the initial structure. For explicit system, the A, B, and C regions are well-separated from each other by the intermediary region. However, for implicit system, the A, B, and C are not well-separated due to the shrinkage of the projection domain. It takes up to 56% strain to unfold and get separated from each other for the regions. This is expected because it is already substantiated that during equilibration the projection domain faces shrinkage of ~57.2% of its length. After that, it can be observed that all the A, B, and C regions as well as intermediary regions reach "significant unfolding" state for both explicit and implicit system. This structural evolution analysis depicts that both systems predict the similar mechanical behavior after their orientations become similar after a certain stretch (~56%).

The only issue remaining is the difference between the stress-strain values in the systems with explicit vs implicit solvation. This discrepancy can be resolved if it is considered that in implicit system, the solvent-surface charged layer interaction is absent. However, the internal stress component can be obtained from the surface force. If it is considered that the tau protein single strand radius is approximately 0.5nm [18], then the area on which the surface force is acting can be calculated. The area multiplied by the surface tension will be equal to the surface force at static equilibrium. Therefore, we can write,

$$F = 2\pi r\gamma = -\sigma_x(\pi r^2), \text{or } \sigma_x = -\frac{2\gamma}{r} \qquad \textbf{(A2.1)}$$

Now, despite having significant charge difference along the filament length, the tau protein surface interacts with weakly hydrophilic MT surface, and therefore, the surface tension of tau protein surface can be approximated with that of water, which is 0.072N/m at 310K. Putting this value in the above equation, it can be calculated that σ_x=-288MPa, which is ignored in the implicit solvation system. After adding this amount of stress with the stress values calculated for the implicit system and then plotting with the explicit system values, it can be observed that the stress values are remarkably close (Figure S7). The slight difference can certainly be attributed to the difference of conformation of the projection domain. Therefore, it can be concluded that if the surface force is included to the calculation, both explicit and implicit systems will show nearly the same stress-strain relation.

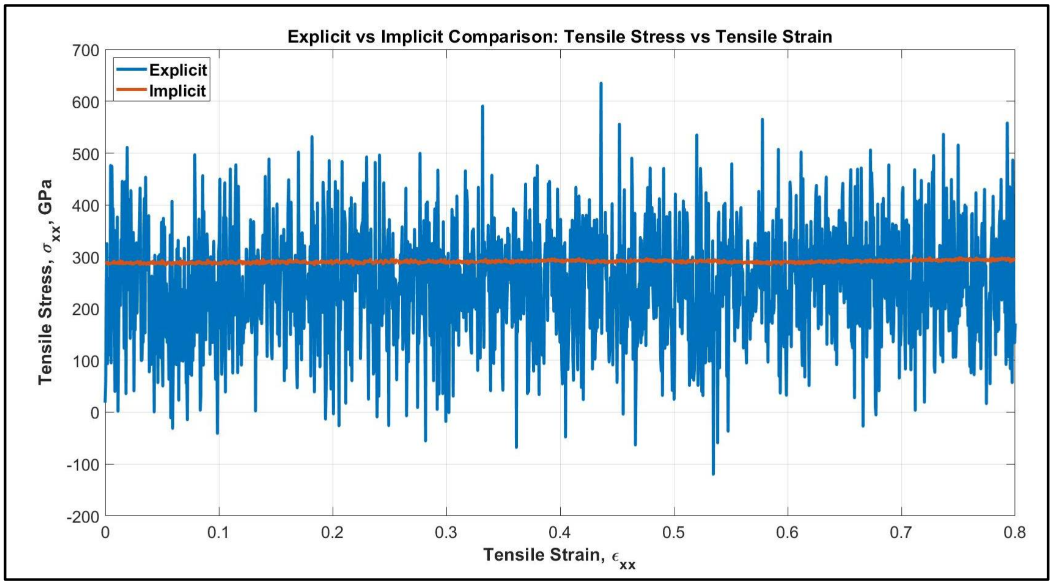

Figure S7: Comparison between the explicit and implicit system stress-strain relation after considering the correction originated from calculating the surface force.

Therefore, despite the constraints that must be dealt with in molecular dynamics simulation, the implicit simulations for single tau protein, dimerized tau protein, and tau-MT interaction systems are providing important insight regarding the behavior of tau protein from strictly mechanical viewpoint which is relevant from traumatic brain injury (TBI) perspective, and the first of its kind. The difference between the explicit and implicit system behaviors can be compensated by including the surface force to the calculation. To the best knowledge of the authors, this work has particularly shed light on strain-rate dependent behavior of tau protein in polymerized state, and highlighted different damage mechanisms in sub-axonal level, which were not explored entirely in earlier studies.

Supplementary Material Part 3

i-TASSER Predicted Models: Reliability of Independently Predicted Structures

When an amino acid sequence is submitted to i-TASSER server, it produces five (5) models with reliability score at descending order. It is relevant to determine to test the stability of independently predicted models under the same equilibration and tensile condition to determine whether a particular model with a particular reliability score is

representative. We have, therefore, compared the potential energy vs time for equilibration of two models of single tau protein (Model 1 and Model 2, both with C-score of -0.03). Later, we have carried out tensile test to find out whether the two models show similar trend of tensile stress vs strain (up to 80% strain, at applied strain rate = $1x10^9s^{-1}$). If in both cases they show similar values, we can be sure that independently predicted structures with similar C-score are similar, both with respect to stability and mechanical behavior. Fig. S8 and S9 depict our finding.

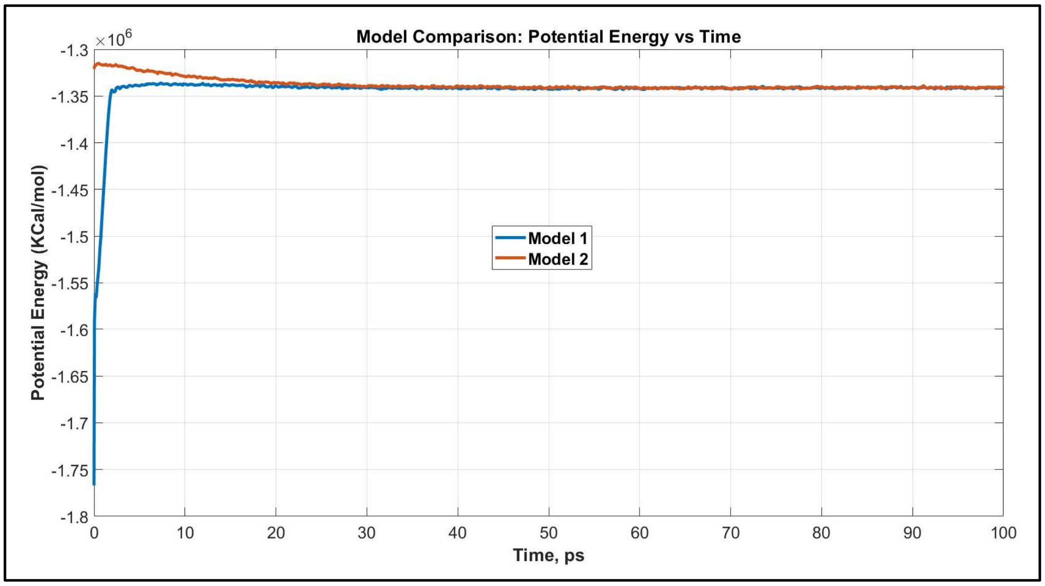

Figure S8: Potential energy vs time for two models. We find that potential energy for both models converge towards similar value over time.

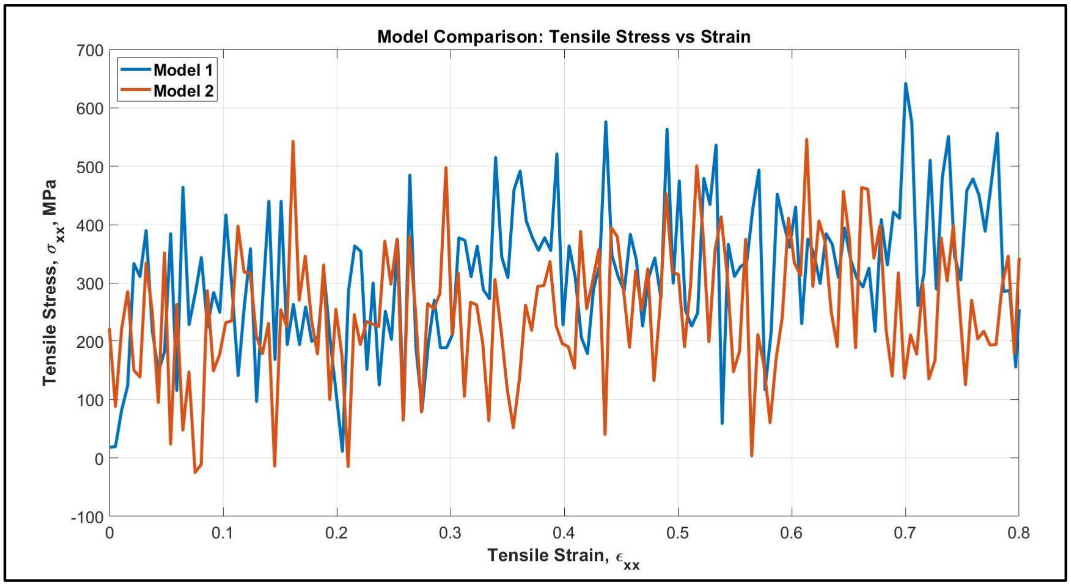

Figure S9: Tensile stress vs strain for two models. We find that they show similar trend after initial discrepancies. The slight variation can be attributed to unconstrained movement in the space. Linear fit shows that for up to 80% strain, the unfolding stiffness for Model 1 is ~227MPa, while for Model 2 is ~126MPa, which are very comparable.

From Fig. S8 and Fig. S9, it is evident that two independently predicted models of single tau protein show similar stability and mechanical behavior, which strengthens the reliability of the C-score of our predicted model and justifies the modeling scheme used in the study.

Supplementary Material Part 4

Tau Protein: Amino Acid Sequence for Isoform F (the most prevalent human tau isoform) [19]

Entire Sequence:

MAEPRQEFEVMEDHAGTYGLGDRKDQGGYTMHQDQEGDTDAGLKESPLQTPTEDGSEEPG

SETSDAKSTPTAEDVTAPLVDEGAPGKQAAAQPHTEIPEGTTAEEAGIGDTPSLEDEAAG

HVTQARMVSKSKDGTGSDDKKAKGADGKTKIATPRGAAPPGQKGQANATRIPAKTPPAPK

TPPSSGEPPKSGDRSGYSSPGSPGTPGSRSRTPSLPTPPTREPKKVAVVRTPPKSPSSAK

SRL **QTAPVPMPDLKNVKSKIGSTENLKHQPGGGKV** **QIINKKLDLSNVQSKCGSKDNIKHV**

PGGGS **VQIVYKPVDLSKVTSKCGSLGNIHHKPGGGQ** **VEVKSEKLDFKDRVQSKIGSLDNI**

THVPGGGN KKIETHKLTFRENAKAKTDHGAEIVYKSPVVSGDTSPRHLSNVSSTGSIDMV

DSPQLATLADEVSASLAKQGL

Note: There are 3 regions in the sequence: projection domain, microtubule (MT) binding domain, and tail. The MT binding domain consists of 4 repeats (block and underlined in the above sequence):

1. QTAPVPMPDLKNVKSKIGSTENLKHQPGGGK,
2. VQIINKKLDLSNVQSKCGSKDNIKHVPGGGS,
3. VQIVYKPVDLSKVTSKCGSLGNIHHKPGGGQ, and
4. VEVKSEKLDFKDRVQSKIGSLDNITHVPGGGN.

The region before the first MT binding domain is the projection domain, and the region after the last MT binding domain is the tail domain.

References

1. Zhang, Y. I-TASSER server for protein 3D structure prediction. *BMC Bioinformatics* **2008**, *9*, 40.

2. Cozzetto, D.; Kryshtafovych, A.; Ceriani, M.; Tramontano, A. Assessment of predictions in the model quality assessment category. *Proteins Struct. Funct. Bioinforma.* **2007**, *69*, 175–183.

3. Wu, S.; Zhang, Y. LOMETS: a local meta-threading-server for protein structure prediction. *Nucleic Acids Res.* **2007**, *35*, 3375–3382.

4. Zhang, Y.; Skolnick, J. Automated structure prediction of weakly homologous proteins on a genomic scale. *Proc. Natl. Acad. Sci.* **2004**, *101*, 7594–7599.

5. Zhang, Y.; Skolnick, J. SPICKER: a clustering approach to identify near-native protein folds. *J. Comput. Chem.* **2004**, *25*, 865–871.

6. Zhang, Y.; Skolnick, J. Scoring function for automated assessment of protein structure template quality. *Proteins Struct. Funct. Bioinforma.* **2004**, *57*, 702–710.

7. Falcon, B.; Zivanov, J.; Zhang, W.; Murzin, A.G.; Garringer, H.J.; Vidal, R.; Crowther, R.A.; Newell, K.L.; Ghetti, B.; Goedert, M. Novel tau filament fold in chronic traumatic encephalopathy encloses hydrophobic molecules. *Nature* **2019**, *568*, 420–423.

8. Falcon, B.; Zhang, W.; Murzin, A.G.; Murshudov, G.; Garringer, H.J.; Vidal, R.; Crowther, R.A.; Ghetti, B.; Scheres, S.H.W.; Goedert, M. Structures of filaments from Pick's disease reveal a novel tau protein fold. *Nature* **2018**, *561*, 137–140.

9. Fitzpatrick, A.W.P.; Falcon, B.; He, S.; Murzin, A.G.; Murshudov, G.; Garringer, H.J.; Crowther, R.A.; Ghetti, B.; Goedert, M.; Scheres, S.H.W. Cryo-EM structures of tau filaments from Alzheimer's disease. *Nature* **2017**, *547*, 185.

10. Seidler, P.M.; Boyer, D.R.; Rodriguez, J.A.; Sawaya, M.R.; Cascio, D.; Murray, K.; Gonen, T.; Eisenberg, D.S. Structure-based inhibitors of tau aggregation. *Nat. Chem.* **2018**, *10*, 170.

11. Kleinjung, J.; Fraternali, F. Design and application of implicit solvent models in biomolecular simulations. *Curr. Opin. Struct. Biol.* **2014**, *25*, 126–134.

12. Chen, J.; Brooks III, C.L.; Khandogin, J. Recent advances in implicit solvent-based methods for biomolecular simulations. *Curr. Opin. Struct. Biol.* **2008**, *18*, 140–148.

13. Chau, M.-F.; Radeke, M.J.; de Inés, C.; Barasoain, I.; Kohlstaedt, L.A.; Feinstein, S.C. The microtubule-associated protein

tau cross-links to two distinct sites on each α and β tubulin monomer via separate domains. *Biochemistry* **1998**, *37*, 17692–17703.

14. SERRANO, L.; MONTEJO DE GARCINI, E.; HERNÁNDEZ, M.A.; AVILA, J. Localization of the tubulin binding site for tau protein. *Eur. J. Biochem.* **1985**, *153*, 595–600.

15. Silber, J.; Cotton, J.; Nam, J.-H.; Peterson, E.H.; Grant, W. Computational models of hair cell bundle mechanics: III. 3-D utricular bundles. *Hear. Res.* **2004**, *197*, 112–130, doi:https://doi.org/10.1016/j.heares.2004.06.006.

16. Rosenberg, K.J.; Ross, J.L.; Feinstein, H.E.; Feinstein, S.C.; Israelachvili, J. Complementary dimerization of microtubule-associated tau protein: Implications for microtubule bundling and tau-mediated pathogenesis. *Proc. Natl. Acad. Sci.* **2008**, *105*, 7445–7450.

17. DeLano, W.L. PyMOL 2002.

18. Ruben, G.C.; Iqbal, K.; Grundke-Iqbal, I.; Johnson Jr, J.E. The organization of the microtubule associated protein tau in Alzheimer paired helical filaments. *Brain Res.* **1993**, *602*, 1–13.

19. Clark, L.N.; Poorkaj, P.; Wszolek, Z.; Geschwind, D.H.; Nasreddine, Z.S.; Miller, B.; Li, D.; Payami, H.; Awert, F.; Markopoulou, K. Pathogenic implications of mutations in the tau gene in pallido-ponto-nigral degeneration and related neurodegenerative disorders linked to chromosome 17. *Proc. Natl. Acad. Sci.* **1998**, *95*, 13103–13107.
